# Identification of novel immune correlates of protection against acute bovine babesiosis by superinfecting cattle with *in vitro* culture attenuated and virulent *Babesia bovis* strains

**DOI:** 10.3389/fimmu.2022.1045608

**Published:** 2022-11-18

**Authors:** Reginaldo G. Bastos, Jacob M. Laughery, Sezayi Ozubek, Heba F. Alzan, Naomi S. Taus, Massaro W. Ueti, Carlos E. Suarez

**Affiliations:** ^1^ Department of Veterinary Microbiology and Pathology, College of Veterinary Medicine, Washington State University, Pullman, WA, United States; ^2^ Department of Parasitology, Faculty of Veterinary Medicine, University of Firat, Elazig, Turkey; ^3^ Parasitology and Animal Diseases Department, Veterinary Research Institute, National Research Center, Dokki, Giza, Egypt; ^4^ Animal Disease Research Unit, United States Department of Agricultural - Agricultural Research, Pullman, WA, United States

**Keywords:** bovine babesiosis, *Babesia bovis*, vaccine, *Babesia* immunity, *Babesia* pathogenesis, *Babesia* immune correlates of protection

## Abstract

The apicomplexan tickborne parasites *Babesia bovis* and *B. bigemina* are the major causative agents of bovine babesiosis, a disease that negatively affects the cattle industry and food safety around the world. The absence of correlates of protection represents one major impediment for the development of effective and sustainable vaccines against bovine babesiosis. Herein we superinfected cattle with attenuated and virulent strains of *B. bovis* to investigate immune correlates of protection against acute bovine babesiosis. Three 6-month-old Holstein calves were infected intravenously (IV) with the *in vitro* culture attenuated Att-S74-T3Bo *B. bovis* strain (10^6^ infected bovine red blood cells (iRBC)/calf) while three age-matched Holstein calves were inoculated IV with normal RBC as controls (10^6^ RBC/calf). All Att-S74-T3Bo-infected calves showed a significant increase in temperature early after inoculation but recovered without treatment. Att-S74-T3Bo-infected calves also developed: (a) monocytosis, neutropenia, and CD4^+^ lymphopenia in peripheral blood on days 3 to 7 post-inoculation; (b) significant levels of TNFα, CXCL10, IFNγ, IL-4, and IL-10 in sera at day 6 after infection; and (c) IgM and IgG against *B. bovis* antigens, starting at days 10 and 30 post-inoculation, respectively. At 46 days post-Att-S74-T3Bo inoculation, all experimental calves were infected IV with the homologous virulent *B. bovis* strain Vir-S74-T3Bo (10^7^ iRBC/calf). All Att-S74-T3Bo-infected calves survived superinfection with Vir-S74-T3Bo without displaying signs of acute babesiosis. In contrast, control animals showed signs of acute disease, starting at day 10 post-Vir-S74-T3Bo infection, and two of them were humanely euthanized at days 13 and 14 after inoculation due to the severity of their symptoms. Also, control calves showed higher (P<0.05) parasite load in peripheral blood compared to animals previously exposed to Att-S74-T3Bo. No significant alterations in the profile of leukocytes and cytokines were observed in Att-S74-T3Bo-inoculated after Vir-S74-T3Bo infection. In conclusion, data demonstrate novel changes in the profile of blood immune cells and cytokine expression in peripheral blood that are associated with protection against acute bovine babesiosis. These identified immune correlates of protection may be useful for designing effective and sustainable vaccines against babesiosis in cattle.

## Introduction

Bovine babesiosis is an economically important tickborne disease of cattle caused by apicomplexan hemoparasites of the genus *Babesia* (1). *Babesia bovis* and *B. bigemina* are the primary causative agents of the disease, and it is estimated that more than 500 million cattle are at risk of contracting the disease in tropical and temperate endemic regions of the world (2–6). Pathogenesis of acute bovine babesiosis is characterized by severe anemia, fever, anorexia, and prostration, which may lead to death or establishment of persistent infection in animals that survive the acute phase of the disease. Acute *B. bovis* infection can also be presented by adhesion of infected red blood cells (iRBC) in the capillaries of the brain, liver, and lungs, among other vital organs, a condition that resembles severe malaria, resulting in high mortality (7, 8). Acaricides to decrease tick infestation, anti-babesicidal drugs, and live attenuated *B. bovis* and *B. bigemina* vaccines are the currently available control strategies against bovine babesiosis. The use of acaricides can select for resistant tick populations and cause toxicity for animals and environment (9). Therapeutic drugs are expensive, unpractical for large herds of cattle, and can also potentially lead to drug resistant parasites (10–12). Live attenuated vaccine strains are relatively efficient but are only used in some endemic areas due to several constraints, including: (a) variable parasite attenuation for naïve adult animals and, consequently, the utilization of these vaccines is usually restricted to young calves (<1-year old); (b) risk to transmit contaminating blood-borne pathogens; (c) the need of *Babesia*-naïve calves to produce the attenuated vaccine strains; (d) potential risk for reversion of virulence; (e) vaccinated animals become carriers/reservoirs of *Babesia* and a potential source for parasite tick acquisition/transmission; (f) vaccine batch variation with implications on quality control; and (g) the need for a cold chain for vaccine distribution and administration (1, 13–15). Combined, these factors clearly illustrate that more efficient and sustainable strategies to control bovine babesiosis are urgently needed.

It is well established that young cattle (<6 months of age) are more resistant to acute bovine babesiosis than adults (>1-year old animals) (16–18). The increased resistance of young animals to *Babesia* infection is spleen-dependent and associated with early activation of innate immune responses (19–22). Specifically, protection against *B. bovis* is correlated with the development of a pro-inflammatory phenotype that is initiated by splenic myeloid cells, such as monocytes and dendritic cells, which induces lymphocytes to produce IFNγ (19, 21, 23). Considering the role of splenic immune cells during *B. bovis* infection, delay in producing IFNγ with concomitant expression of IL-10 is associated with progression of acute disease (19, 20). Interestingly, cattle that survive the acute phase of babesiosis caused by *B. bovis* become chronically infected and reservoirs for tick acquisition but are protected against clinical disease upon reinfection with related parasite strains (1, 14, 24, 25). Collectively, these previous observations demonstrate that protective immunity against *Babesia* parasites is achievable, and a better understanding of this mechanism could help towards developing effective vaccines against bovine babesiosis.

Following the genome sequencing of the *B. bovis* T2Bo strain (26), a marked increase in understanding the parasite’s biology has emerged from functional studies using genetically modified parasites and high-throughput approaches (27–32). Therefore, several novel parasite blood and tick stage antigens have been discovered and evaluated in vaccine trials (31, 33–35). Availability of genome sequences and development of genetic tools for *B. bovis* also allowed the use of transfected parasites as a delivery system for tick vaccine antigens (36, 37). Despite the progress, an efficient subunit vaccine against *B. bovis*, or any other *Babesia* spp., has not yet been achieved. This failure is associated in part with the absence of well-defined immune correlates of protection, which represents an important knowledge gap that has hampered the development of effective and sustainable vaccines against babesiosis.

To address the knowledge gap on correlates of immune protection against *B. bovis*, in this study, we investigated the immune responses of cattle infected with the *in vitro* culture attenuated non-tick transmissible *B. bovis* S74-T3Bo strain (Att-S74-T3Bo) (37, 38). Development of protective immunity was assessed by superinfecting Att-S74-T3Bo-inoculated animals with the homologous virulent *B. bovis* S74-T3Bo strain (Vir-S74-T3Bo). Att-S74-T3Bo-infected calves developed an early significant monocytosis, neutropenia and CD4^+^ lymphopenia in peripheral blood. In addition, these animals showed a balanced profile of pro- and anti-inflammatory cytokines in peripheral blood and were fully protected against infection with Vir-S74-T3Bo. By focusing on peripheral blood, herein we identified novel immune correlates of protection that may have implications for the development of anti-*B. bovis* vaccines.

## Material and methods

### *B. bovis* strains

The *B. bovis* strain S74-T3Bo, which originated from the parental T2Bo strain (26), was used in this study. A batch of the S74-T3Bo strain has been maintained in culture, under standard conditions, as previously described (39), for more than 10 years. This strain has been shown to be attenuated for cattle and non-tick transmissible (37, 38), and consequently, termed Att-S74-T3Bo in this present study. Stabilates with the homologous virulent S74-T3Bo strain (Vir-S74-T3Bo), derived from experimentally infected cattle, were used to infect calves, as described in previous studies (19, 38, 40).

### Att-S74-T3Bo and Vir-S74-T3Bo infections

A group of three male Holstein calves, 6 months of age, were inoculated intravenously (IV) with Att-S74-T3Bo iRBC (10^6^ iRBC/calf). Inoculum was prepared from a fresh parasite culture. Another group of three male Holstein calves, 6 months of age, were inoculated IV with 10^6^ normal RBC/calf and served as controls. After inoculation, all animals were monitored daily for signs of acute bovine babesiosis, including temperature, packed cell volume (PCV), *B. bovis* load in peripheral blood, and behavioral alterations. At day 46 after Att-S74-T3Bo inoculation, infected and control calves were infected IV with 10^7^ Vir-74-T3Bo iRBC. This parasite dose has been shown to induce severe acute disease in cattle in previous studies (40–43). Following Vir-74-T3Bo infection, all calves were monitored daily for clinical signs of acute bovine babesiosis, as described above. All animal experiments were approved by the University of Idaho Institutional Animal Care and Use Committee (IACUC protocol number 2020-51).

### *B. bovis* real-time quantitative PCR

Parasite load in peripheral blood of animals after infection was measured by real-time quantitative PCR (qPCR), as previously described (42). Briefly, peripheral blood from experimental animals was collected *via* jugular venipuncture into Vacutainer^®^ tubes containing ethylenediamine tetra acetic acid (EDTA) (BD Company, Franklin Lakes, NJ) at several time-points after infection. Total genomic DNA from whole blood was extracted using the QIAamp^®^ DNA Blood Mini Kit (QIAGen, Valencia, CA) following the manufacture’s protocol. Genomic DNA was used for qPCR to amplify the single copy *B. bovis msa-1* gene. For the *msa-1* qPCR, specific primers (5’ gatgcgtttgcacatgctaag 3’ and 5’ cgggtacttcggtgctctca 3’) and probe (FAM 5’-cacgctcaagtaggaaattttgttaaacctgga-3’ TAMRA) were used following the assay conditions described elsewhere (42).

### Indirect ELISA to *B. bovis* rhoptry associated protein 1 C-terminal segment

The presence of antibodies in serum of infected animals against the C-terminal segment of the immunodominant antigen rhoptry associated protein 1 (RAP-1 CT) was evaluated by indirect ELISA (iELISA), as previously described (44). Briefly, peripheral blood was collected in Vacutainer^®^ tubes with no anticoagulant, and serum samples were obtained by standard procedures. For iELISA, 96-well Immulon™ 2HB microtiter plates (Thermo Fisher Scientific, Waltham, MA) were coated overnight at 4 °C with 50 µl of recombinant *B. bovis* RAP-1 CT (2 µg/ml). Plates were then washed three times using 200 µl blocking buffer (0.2% I-Block™ in 1xPBS with 0.1% Tween 20) and blocked with 300 µl of the same buffer for one hour at 30 °CC. After blocking, bovine serum samples were diluted 1/10 in blocking buffer, and 50 µl was added to triplicate individual wells. Plates were incubated for one hour at 30 °CC and then washed five times in 200 µl blocking buffer. After that, 50 µl of a 1/1000 dilution of anti-bovine total IgG, IgG1, IgG2, or IgM peroxidase labeled secondary antibodies (SeraCare, Milford, MA) were added to each well, and plates were incubated for 45 minutes at 30 °CC. After incubation, plates were washed four times using 200 µl blocking buffer and two times with 200 µl 1xPBS with 0.1% Tween 20. Fifty-five µl of SureBlue™ TMB (SeraCare, Milford, MA) was then added to each well and plates were incubated for 10 minutes. Reaction was stopped by adding 55 µl TMB stop solution (SeraCare, Milford, MA), and absorbance was measured at 450 nm using the SpectraMax^®^ 190 plate reader (Molecular Devices, San Jose, CA). Average values with standard deviations were then calculated and plotted to a chart.

### Immunoblot

The presence of anti-*B. bovis* antibodies after infection was also examined by immunoblot. Briefly, total antigens (2 µg/lane) from uninfected bovine RBC, Att-S74-T3Bo iRBC (2% percentage of parasitized erythrocytes, PPE), or Vir-S74-T3Bo iRBC (2% PPE) were ran through 4-20% TGX™ gels (Bio-Rad Laboratories, Hercules, CA). Antigens were then transferred to nitrocellulose membranes using iBlot 2™ (Invitrogen, Waltham, MA), and the membranes were blocked in 5% milk overnight. Membranes were then washed one time in 1xPBS with 0.1% Tween 20 (PBS-T) and incubated with a 1/50 dilution of bovine serum in 5% milk for one hour rocking at 30 °CC. After that, membranes were washed three times using PBS-T and incubated with a 1/1000 dilution of anti-bovine IgG peroxidase labeled secondary antibodies (SeraCare, Milford, MA) in 5% milk for 30 minutes rocking at 30 °CC. Membranes were then washed again three times in PBS-T and sprayed with WesternBright^®^ ECL-spray (Advansta, San Jose, CA). Visualization of immune complexes was performed using the Azure™ Imaging System (Azure Biosystems, Dublin, CA).

### Blood cell count

Cell blood count was evaluated in all experimental animals after *B. bovis* infection using the ProCyte One™ Hematology Analyzer (IDEXX Laboratories, Westbrook, ME). Peripheral blood from the experimental animals was collected at several time-points after infection, as described above into Vacutainer^®^ tubes containing EDTA (BD Company, Franklin Lakes, NJ). After collection, whole blood samples were homogenized for 5 minutes and the numbers of total leukocytes, lymphocytes, monocytes, neutrophils, RBC, and reticulocytes were measured. Results of each leukocyte population are presented as 1,000 cells/μl of blood, and RBC and reticulocyte are shown as 1,000,000 cells/μl of blood.

### Phenotype of peripheral blood mononuclear cells by flow cytometric analysis

Peripheral blood from all experimental animals was collected at several time-points after infection in Vacutainer^®^ tubes containing EDTA. Peripheral blood mononuclear cells (PBMC) were isolated using Histopaque^®^-1077 (Sigma, St. Louis, MO), as per a standard protocol. Cells were then labeled with monoclonal antibodies to surface markers (Table S1), followed by secondary antibodies (Table S2) using standard flow cytometry protocols, as previously described (45). Flow cytometric analysis was performed using the Guava^®^ easyCyte™ flow cytometer (Luminex, Austin, TX), and data were acquired using the InCyte™ guavaSoft™ software version 3.1.1 (Luminex, Austin, TX). A single-color panel was used for phenotyping CD14^+^ monocytes, CD335^+^ NK cells, CD4^+^ T cells, CD8^+^ T cells, γδ T cells, and B cells in PBMC. A minimum of 20,000 events were collected for each cell population. After acquisition, data were analyzed using FCS Express™, version 6 (DeNovo™ Software, Pasadena, CA). Results are presented as percentage of target cell populations in PBMC.

### Cytokine measurements

Concentration of cytokines in serum from infected animals was measured by ELISA. Bovine IFNγ was assessed by a single-metabolite assay (Bio-Rad Laboratories, Hercules, CA) following the manufacture’s protocol, using the SpectraMax^®^ 190 plate reader (Molecular Devices, San Jose, CA). Results for IFNγ are presented as pg/ml and minimal detectable level was 25 pg/ml. Bovine IL-1β, IL-4, IL-6, IL-8, IL-10, CXCL10, and TNFα were evaluated by MILLIPLEX^®^ Bovine Cytokine/Chemokine Magnetic Bead (Millipore-Sigma, Burlington, MA) following the manufacture’s protocol. Minimal detectable levels were as follows: 0.1 pg/ml IL-1β, 12.8 pg/ml IL-4, 2.6 pg/ml IL-6, 2.2 pg/ml IL-8, 0.96 pg/ml IL-10, 0.6 pg/ml CXCL10, and 12.8 pg/ml TNFα. Standard curves and quality controls for each cytokine in the multiplex assay were prepared following the manufacture’s protocol. The LX-200™ instrument (Luminex, Austin, TX) was used for the multiplex analysis. Data acquisition was done by xPONENT^®^ software (Luminex, Austin, TX), and results were analyzed by Belysa^®^ software (Millipore-Sigma, Burlington, MA).

### Data analysis

Graphpad Prism software version 9 (GraphPad Software, San Diego, CA) was used for data analysis and to generate graphs. Results of temperature, PCV, parasite load, percentage of cells in blood, percentage of PBMC, and cytokines in serum are shown as mean and standard deviation of experimental and control samples. Means were compared with a two-tailed *t* test, and a P value of <0.05 was considered significant.

## Results

### Att-S74-T3Bo *B. bovis* infection of cattle

**Figure 1:**
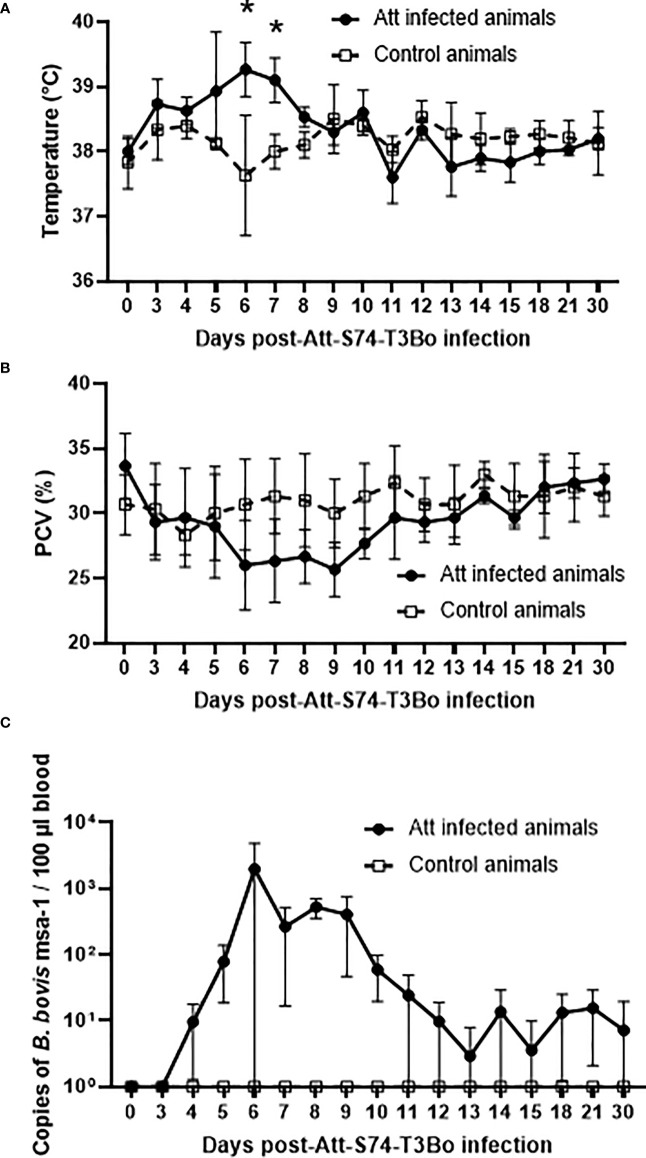
Temperature **(A)**, packed cell volume (PCV) **(B)**, and *B. bovis* load in peripheral blood accessed by real-time quantitative PCR **(C)** after infection with the *in vitro* culture attenuated *B bovis* strain Att-S74-T3Bo. Att infected animals (n=3). Control animals (n=3). * P<0.05.

We have previously shown that the Att-S74-T3Bo *B. bovis* strain has an attenuated phenotype in cattle, and it is not tick transmissible (37, 38). However, no previous experiments have been performed to investigate the ability of this strain to induce a protective immune response in cattle against experimental infection with a virulent *B. bovis* strain. Therefore, as a starting point of our study, we systematically defined the characteristics of the Att-S74-T3Bo acute infection in cattle. A group of three calves was inoculated IV with 10^6^ iRBC and monitored daily for signs of acute bovine babesiosis. Att-S74-T3Bo-inoculated animals showed mild but significant (P<0.05) elevation of temperature on days 6 (39.2 °CC [±0.41]) and 7 (39.1 °CC [±0.34]) post-infection compared to uninfected control calves (**Figure 1A**). Concomitantly, infected animals had decreased PCV on days 6 to 10 after infection; however, no statistical significance was observed compared to control animals (**Figure 1B**). Peak of parasite load (approx. 5x10^3^ parasites/100 µl of blood) occurred at day 6 after Att-S74-T3Bo inoculation. Parasite DNA was then intermittently detected from days 12 to 30 post-inoculation (**Figure 1C**). Despite the increase in temperature, drop in PCV, and parasitemia, the Att-S74-T3Bo-infected calves did not develop additional signs of acute babesiosis, such as anorexia and neurological symptoms, and were able to control acute infection, transitioning to subclinical infection without the administration of anti-pyretic or babesicidal therapeutics. Collectively, results confirmed that the Att-S74-T3Bo *B. bovis* strain establishes infection in calves, which is characterized by moderate parasite replication in peripheral blood, significant increase in temperature, and marginal drop in PCV.

### Cattle infected with Att-S74-T3Bo *B. bovis* are protected against Vir-S74-T3Bo

**Figure 2:**
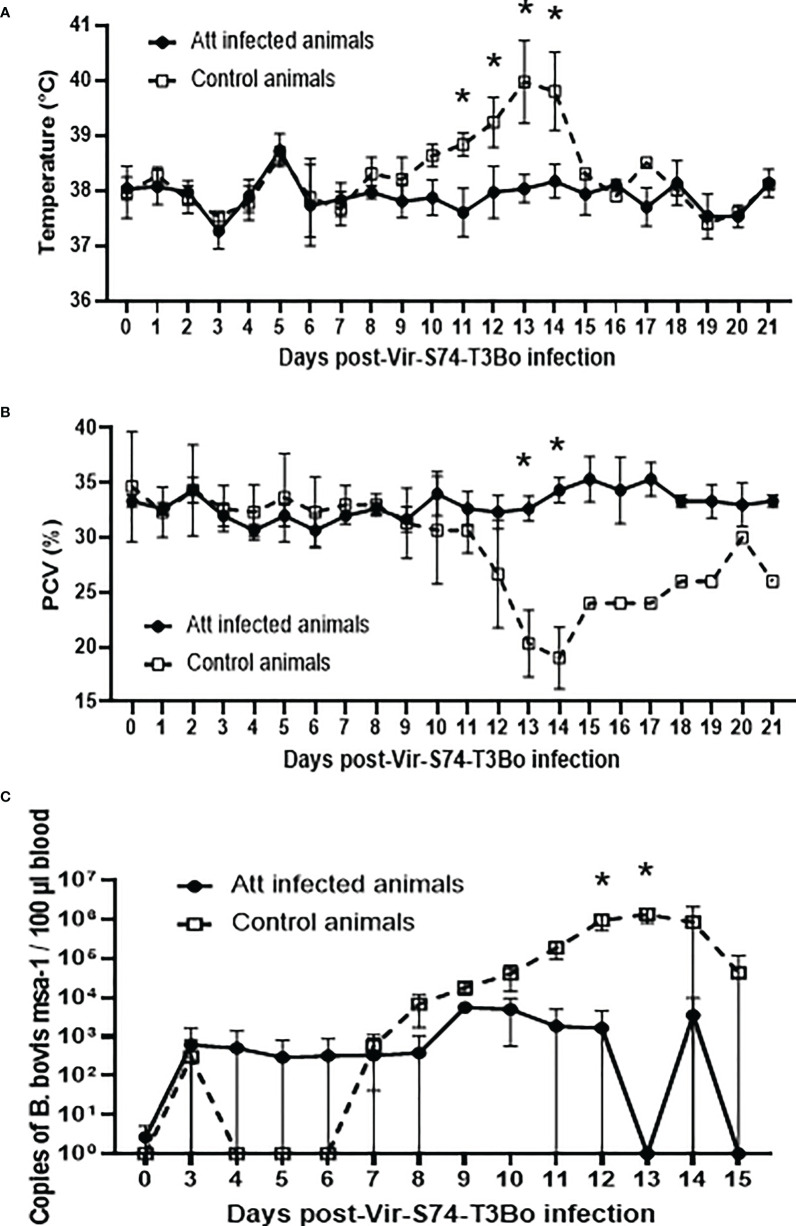
Temperature **(A)**, packed cell volume (PCV) **(B)**, and *B. bovis* load in peripheral blood accessed by real-time quantitative PCR **(C)** after infection with the virulent *B bovis* strain Vir-S74-T3Bo. Att infected animals (n=3). Control animals (n=3; two animals of the control group were humanely euthanized at days 13 and 14 after Vir-S74-T3Bo inoculation due to the severity of the disease). * P<0.05.

After demonstrating that Att-S74-T3Bo infected calves without causing severe acute disease, we further investigated whether these animals were protected against the homologous virulent strain Vir-S74-T3Bo. To test this proposition, Att-S74-T3Bo-inoculated and control calves were infected IV with 10^7^ Vir-S74-T3Bo iRBC at day 46 after the Att-S74-T3Bo infection. All animals were monitored daily for clinal signs of acute bovine babesiosis and parasite load in peripheral blood. A significant increase (P<0.05) in temperature, combined with a marked drop in PCV, was observed in all three control animals starting at day 11 after Vir-S74-T3Bo infection compared to the calves previously inoculated with Att-S74-T3Bo (**Figures 2A, B**). Following the regulations of the approved IACUC protocol, two calves in the control group were humanely euthanized at days 13 and 14 after Vir-S74-T3Bo infection due to the severity of the acute disease. Temperature of the control calf that did not succumb to acute disease returned to physiological levels around day 16 post-infection; however, the animal’s PCV values remained low until day 21 after infection (**Figure 2B**). No significant alterations in temperature and PCV were observed in the calves previously infected with Att-S74-T3Bo (**Figures 2A, B**). Parasite load in Att-S74-T3Bo-infected calves reached a peak of 5x10^3^ copies of *B. bovis msa-1*/100 µl of blood at day 9 after Vir-S74-T3Bo superinfection, and then it was intermittently detected until 45 days post-superinfection when the experiment was terminated (**Figure 2C**). In contrast, control animals showed a significant increase (P<0.05) in parasite load starting at day 10 after Vir-S74-T3Bo infection, which peaked at day 13 post-infection (approximately 10^6^ copies of *B. bovis msa-1*/100 µl of blood). The control calf that survived acute disease exhibited a decrease in parasite load to approximately 10^4^-10^5^ copies of *B. bovis msa-1*/100 µl of blood, starting at day 15 post-Vir-S74-T3Bo infection (**Figure 2C**). Altogether, results demonstrate that calves previously infected with Att-S74-T3Bo were fully protected against the virulent homologous strain Vir-S74-T3Bo.

### Alterations in peripheral blood cells of *B. bovis*-protected cattle

**Figure 3:**
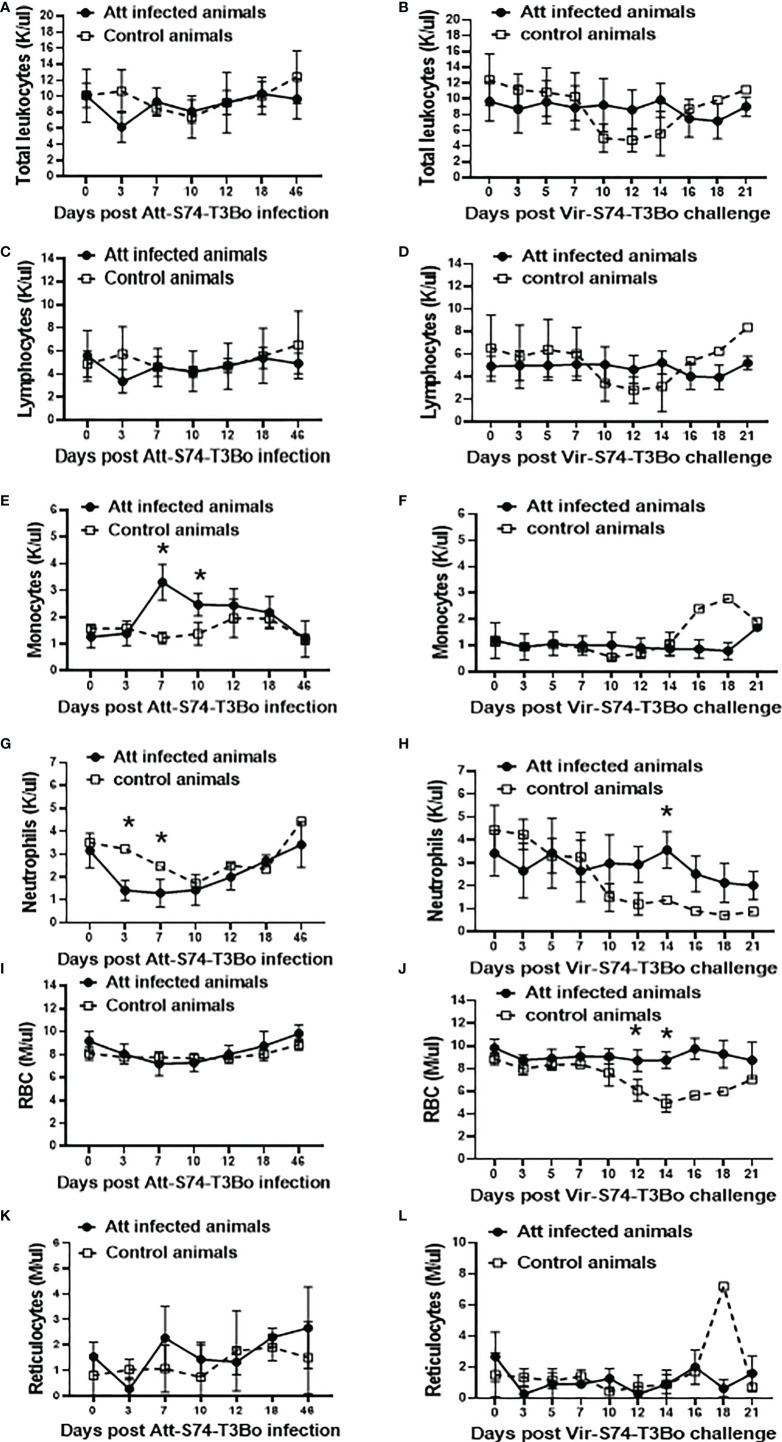
Absolute numbers of total leukocytes **(A, B)**, lymphocytes **(C, D)**, monocytes **(E, F)**, neutrophils **(G, H)**, red blood cells (RBC) **(I, J)**, and reticulocytes **(K, L)** after infections with the *in vitro* culture attenuated *B*. *bovis* strain Att-S74-T3Bo and the virulent *B. bovis* strain Vir-S74-T3Bo. Att infected animals (n=3). Control animals (n=3; two animals of the control group were humanely euthanized at days 13 and 14 after Vir-S74-T3Bo inoculation due to the severity of the disease). * P<0.05.

The observation that Att-S74-T3Bo infection protects cattle against a homologous virulent strain prompted us to investigate alterations in blood cells in the protected animals (**Figure 3**). Despite a marginal decreased in total leukocytes and lymphocytes at day 3 after Att-S74-T3Bo infection, and subsequently through days 10 and 14 after Vir-S74-T3Bo infection in control animals, no significant alterations were observed in these cell populations among experimental animals (**Figures 3A–D**). In contrast, data demonstrated a significant (P<0.05) monocytosis on days 7 and 10 post-Att-S74-T3Bo infection (**Figure 3E**). No alterations in monocytes were detected in Att-S74-T3Bo-inoculated calves after superinfection; however, the one control animal that survived Vir-S74-T3Bo infection showed an increase of monocytes starting at day 16 after infection (**Figure 3F**). A significant decrease in neutrophils in peripheral blood was observed starting at day 3 post-Att-S74-T3Bo inoculation, which returned to physiological levels at day 10 post-infection (**Figure 3G**). Neutrophils also decreased significantly in the control animals starting at 12 days after Vir-S74-T3Bo infection, but no alterations were detected in Att-S74-T3Bo-inoculated animals post-Vir-S74-T3Bo superinfection (**Figure 3H**). Att-S74-T3Bo infection did not alter the total number of RBC (**Figure 3I**), confirming our observation of the absence of significant changes in PCV after infection. On the other hand, control animals developed a dramatic and significant decrease in the number of RBC starting at day 12 after Vir-S74-T3Bo infection (**Figure 3J**). No significant changes in the absolute numbers of reticulocytes after infections with Att-S74-T3Bo and Vir-S74-T3Bo were observed, except for the one control calf that survived Vir-S74-T3Bo infection that showed a peak of reticulocytes at day 18 post-infection (**Figures 3K, L**). Combined, these results demonstrate significant alterations of monocytes and neutrophils in peripheral blood starting at day 3 after Att-S74-T3Bo infection. No significant changes were observed in blood cells of the protected Att-S74-T3Bo-inoculated animals after superinfection with Vir-S74-T3Bo. Interestingly, the control calf that did not succumb to Vir-S74-T3Bo infection developed a similar but delayed pattern of alterations for neutrophils and monocytes compared to protected Att-S74-T3Bo-infected animals.

### Phenotypic changes in PBMC following Att-S74-T3Bo and Vir-S74-T3Bo infections

**Figure 4:**
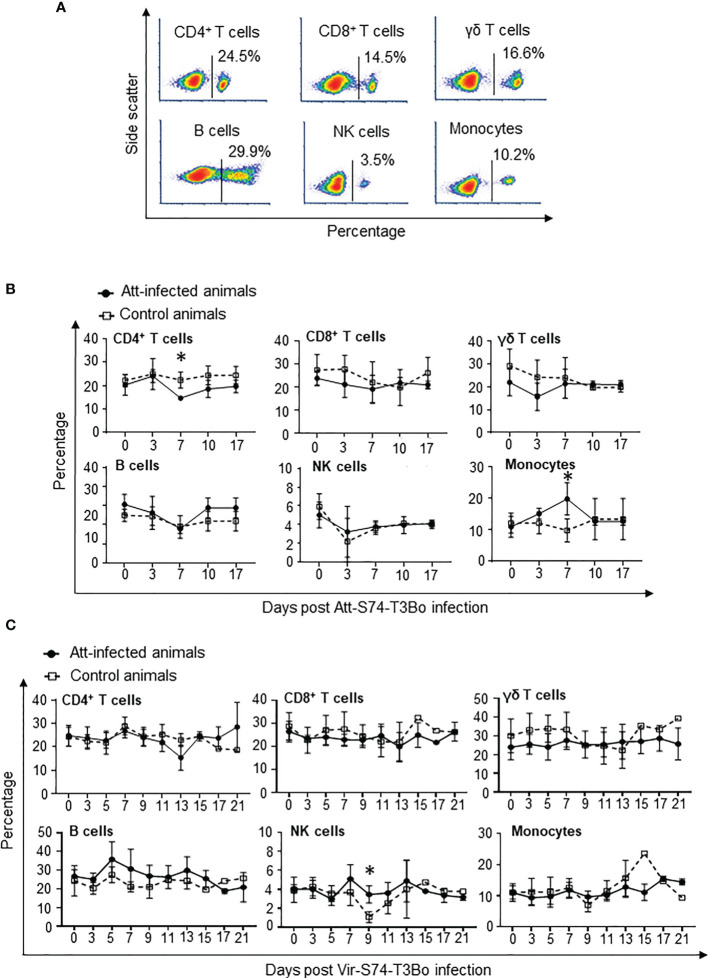
Kinetics of peripheral blood mononuclear cells (PBMC) after infections with the *in vitro* culture attenuated *B. bovis* strain Att-S74-T3Bo and the virulent *B. bovis* strain Vir-S74-T3Bo. Flow cytometric analysis showing PBMC from a normal, uninfected representative calf stained for CD4^+^ T cells, CD8^+^ T cells, γδ T cells, CD14^+^ monocytes, and CD335^+^ natural killer cells (NK) **(A)**. Kinetics of CD4^+^ T cells, CD8^+^ T cells, γδ T cells, B lymphocytes, CD14^+^ monocytes, and CD335^+^ NK cells after Att-S74-T3Bo infection **(B)**. Kinetics of CD4^+^ T cells, CD8^+^ T cells, γδ T cells, B lymphocytes, CD14^+^ monocytes, and CD335^+^ NK cells following Vir-S74-T3Bo infection **(C)**. Att infected animals (n=3). Control animals (n=3; two animals of the control group were humanely euthanized at days 13 and 14 after Vir-S74-T3Bo inoculation due to the severity of the disease). * P<0.05.

After showing significant alterations in monocytes and neutrophils in whole blood of protected calves, we investigated in more details the dynamics of PBMC populations in Att-S74-T3Bo-infected animals following Vir-S74-T3Bo superinfection. To that end, we used flow cytometric analysis to examine the percentage of CD4^+^ T cells, CD8^+^ T cells, γδ T cells, B cells, NK cells, and monocytes in PBMC during acute infection with Att-S74-T3Bo following superinfection with Vir-S74-T3Bo (**Figure 4**). Flow cytometry results of PBMC stained for CD4^+^ T cells, CD8^+^ T cells, γδ T cells, B cells, NK cells, and monocytes from a representative uninfected calf are presented in **Figure 4A**. Calves infected with Att-S74-T3Bo developed a moderate but significant lymphocytopenia of CD4^+^ T cells at day 7 post-infection (**Figure 4B**). No alterations in CD8^+^ T cells, γδ T cells, B cells, and NK cells were observed after Att-S74-T3Bo inoculation (**Figure 4B**). Percentage of monocytes in PBMC increased significantly in Att-S74-T3Bo-infected calves at day 7 post-infection compared to controls (**Figure 4B**), confirming our previous results in whole blood. No significant alterations were detected in CD4^+^ T cells, CD8^+^ T cells, γ™; T cells, B cells, and monocytes after Vir-S74-T3Bo infection (**Figure 4C**). A significant decrease in NK cells at day 9 after Vir-S74-T3Bo infection was observed in control calves in comparison to Att-S74-T3Bo-inoculated animals (**Figure 4C**). As demonstrated in whole blood, the calf that survived Vir-S74-T3Bo infection presented a marked increase of monocytes in PBMC; however, this alteration was delayed compared to the Att-S74-T3Bo-inoculated animals and occurred at day 15 post-Vir-S74-T3Bo infection (**Figure 4C**). In summary, data demonstrated early monocytosis combined with decrease of neutrophils and CD4^+^ T cells in peripheral blood of Att-S74-T3Bo-inoculated calves that were protected against Vir-S74-T3Bo. In addition, the control calf that survived infection did present monocytosis and neutropenia late after Vir-S74-T3Bo inoculation compared to Att-S74-T3Bo-inoculated protected animals.

### Protective immune responses following Att-S74-T3Bo infection

**Figure 5:**
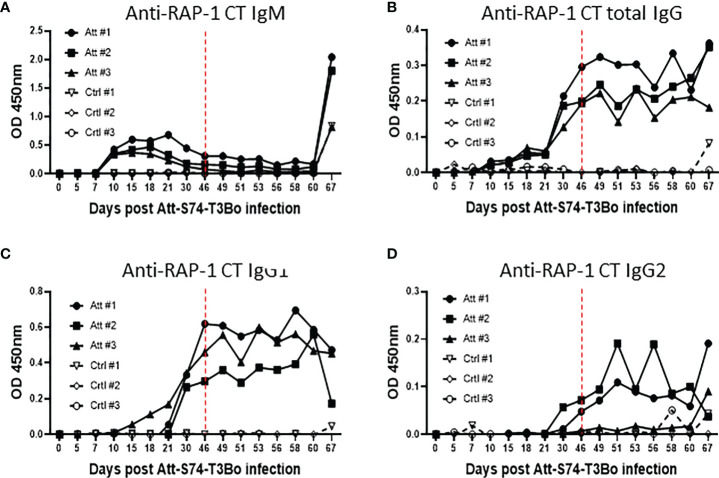
Humoral immune response accessed by indirect ELISA using the immunodominant antigen rhoptry associated protein 1 C-terminal (RAP-1 CT) after infections with the *in vitro* culture attenuated *B. bovis* strain Att-S74-T3Bo and the virulent *B. bovis* strain Vir-S74-T3Bo. Anti-RAP-1 CT IgM **(A)**. Total anti-RAP-1 CT IgG **(B)**. Anti-RAP-1 CT IgG1 **(C)**. Anti-RAP-1 CT IgG2 **(D)**. Results are presented as optical density (OD) of individual experimental animals. Att, animals infected with Att-S74-T3Bo and superinfected with Vir-S74-T3Bo. Ctrl, control animals infected with Vir-S74-T3Bo. Dashed red lines indicate day zero of Vir-S74-T3Bo infection.

**Figure 6:**
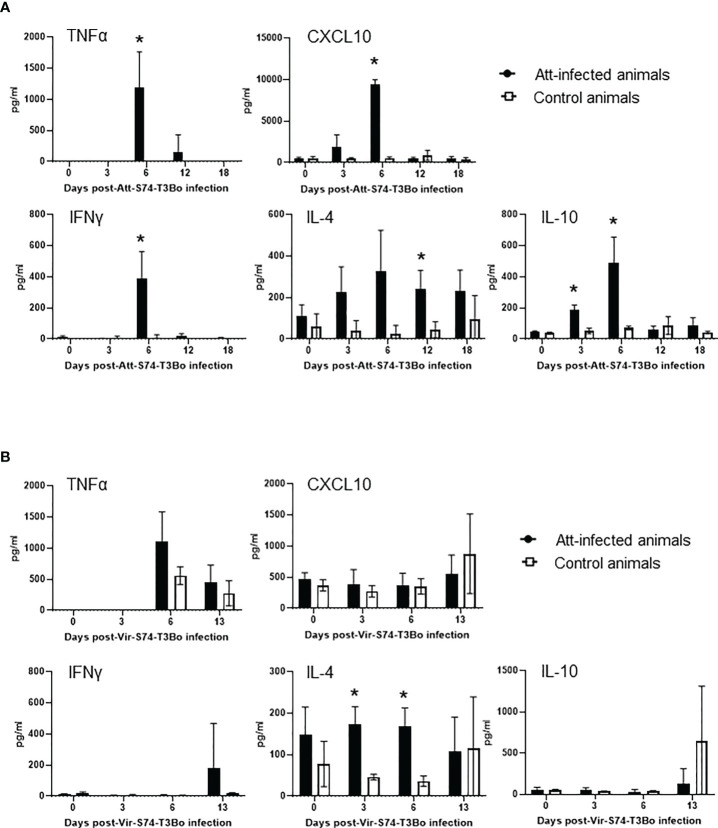
Presence of TNFα, CXCL10, IFNγ, IL-4, and IL-10 in peripheral blood of cattle infected with the *in vitro* culture attenuated *B. bovis* strain Att-S74-T3Bo **(A)** following superinfection with the virulent *B*. *bovis* strain Att-S74-T3Bo **(B)**. Att infected animals (n=3). Control animals (n=3; two animals of the control group were humanely euthanized at days 13 and 14 after Vir-S74-T3Bo inoculation due to the severity of the disease). * P<0.05.

After demonstrating alterations in the profile of blood cells following infections with Att-S74-T3Bo and Vir-S74-T3Bo, we investigated the immune responses induced in Att-S74-T3Bo-inoculated protected animals. Immunoblot results indicated that all three Att-S74-T3Bo-infected animals developed IgG antibodies to parasite antigens (**Figure S1**). Immunoblot data also showed that no antibodies to *B. bovis* antigens were detected in serum of the control calves 14 days after Vir-S74-T3Bo infection, when all three animals were exhibiting marked signs of acute babesiosis and two of them were euthanized due to the severity of the disease (**Figure S1**). In fact, antibodies to parasite antigens were detected in serum of the control calf that survived Vir-S74-T3Bo infection only at day 21 post-inoculation, by the time that acute disease had been resolved. iELISA was performed using the immune dominant *B. bovis* RAP-1 CT as antigen to investigate the level of IgM, total IgG, IgG1, and IgG2 after Att-S74-T3Bo and Vir-S74-T3Bo infections. Data demonstrated detectable levels of anti-RAP-1 CT IgM in all three Att-S74-T3Bo-infected animals starting at 10 days post-infection (**Figure 5A**). After that, the levels of IgM remained marginal and peaked dramatically again at day 21 after Vir-S74-T3Bo superinfection (67 days after Att-S74-T3Bo infection). Levels of total IgG and IgG1 against RAP-1 CT showed a sharp increase starting at day 30 after Att-S74-T3Bo infection, and it remained at those levels until the end of the experiment at 21 days post-Vir-S74-T3Bo superinfection (**Figures 5B, C**). Comparatively, anti-RAP-1 CT IgG2 levels were lower than IgG1, and one Att-S74-T3Bo-infected animal developed only marginal levels of this immunoglobulin subclass (**Figure 5D**). Noteworthy, no boost effect was observed in the levels of anti-RAP-1 CT IgG after Vir-S74-T3Bo superinfection. No presence of IgM, total IgG, IgG1, and IgG2 to RAP-1 CT were detected in control calves in the first 14 days after Vir-S74-T3Bo infection (**Figure 5**), confirming our immunoblot results described above. In fact, the control calf that survived Vir-S74-T3Bo infection developed only minimal levels of IgM and IgG starting at day 21 after inoculation when the symptoms of acute disease had been resolved (**Figures 2**, **3**, **5**). Next, we evaluated the presence of soluble polypeptides for IFNγ, IL-1β, IL-4, IL-6, IL-8, IL-10, CXCL10, and TNFα in serum of animals following infections with Att-S74-T3Bo and Vir-S74-T3Bo (**Figure 6**). Quantitative analysis revealed significant levels of the pro-inflammatory cytokines TNFα, IFNγ, and CXCL10 at day 6 after Att-S74-T3Bo infection compared to uninfected control animals (**Figure 6A**). Also, the Att-S74-T3Bo-infected calves developed significant levels of IL-10 at days 3 and 6 post-infection, and IL-4 at day 12 post-infection (**Figure 6A**). IL-6 and IL-8 were not detected in sera from Att-S74-T3Bo-infected animals (data not shown). No significant differences were found in the levels of TNFα, CXCL10, and IFNγ after infection with Vir-S74-T3Bo, despite the slightly higher concentration of these cytokines in serum from calves previously inoculated with Att-S74-T3Bo compared to controls (**Figure 6B**). No significant levels of IL-10 were detected in Att-S74-T3Bo-infected and control animals after Vir-S74-T3Bo infection, except for an increase of this cytokine in control calves at 13 days post-infection, at the time two control animals were euthanized due to the severity of acute disease (**Figure 6B**). Interestingly, Att-S74-T3Bo-inoculated animals showed significant levels of IL-4 at days 3 and 6 post-Vir-S74-T3Bo infection compared to controls (**Figure 6B**). No detectable levels of IL-6 and IL-8 were found in sera after Vir-S74-T3Bo infection (data not shown). Altogether, these data indicate that Att-S74-T3Bo infection results in the development of protective immune responses in calves characterized by early expression of a balance of inflammatory and regulatory cytokines in peripheral blood, and significant levels of antibodies to *B. bovis* antigen late during infection. In addition, no significant alteration in the levels of IFNγ, IL-1β, IL-4, IL-6, IL-8, IL-10, CXCL10, and TNFα was observed in peripheral blood after Vir-S74-T3Bo infection by comparing Att-S74-T3Bo-inoculated and naïve control animals.

## Discussion

**Figure 7:**
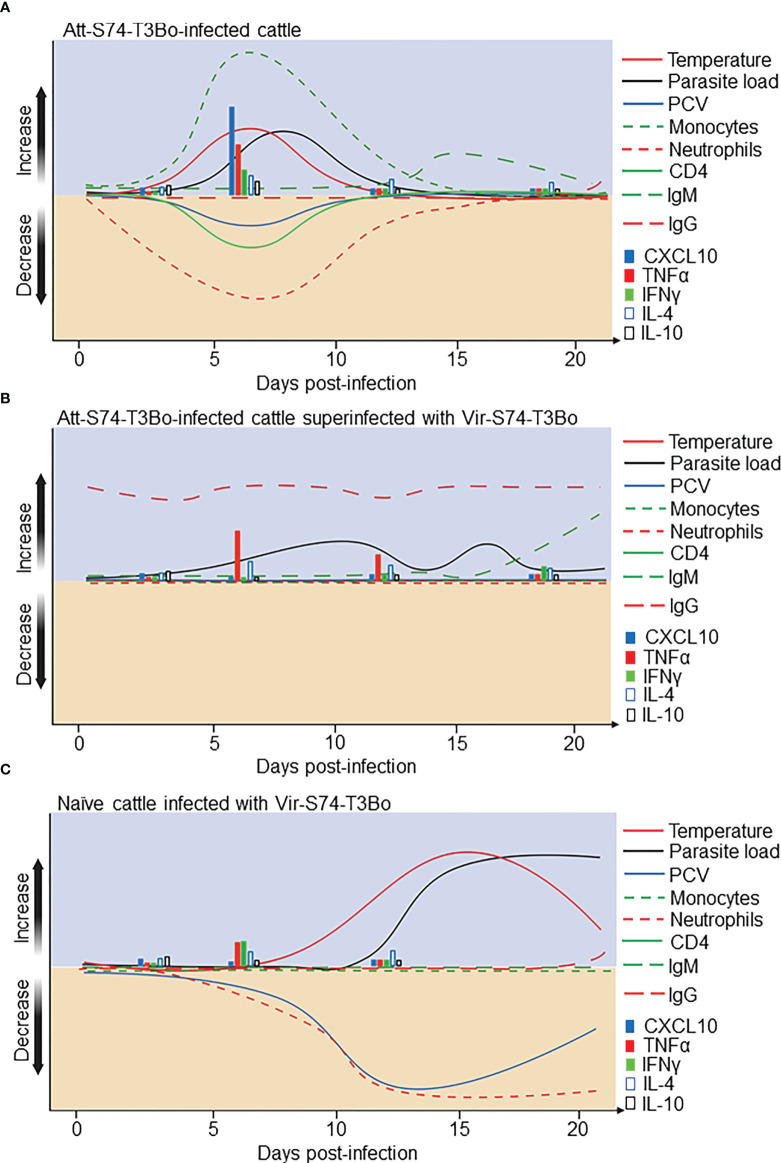
Proposed model for the immunological events after infection with the *in vitro* culture attenuated *B*. *bovis* strain Att-S74-T3Bo and superinfection with the virulent *B*. *bovis* strain Vir-S74-T3Bo. **(A)** Att-S74-T3Bo infection in naïve animals. **(B)** Vir-S74-T3Bo superinfection in Att-S74-T3Bo-protected. **(C)** Vir-S74-T3Bo infection in naïve animals.

The absence of well-defined immune correlates of protection and proper understanding of babesiosis pathogenesis are considered important aspects that hinder the development of effective vaccines against *Babesia* parasites. In this study we addressed these knowledge gaps by using cattle, the natural host of *B. bovis*. By infecting calves with the *in vitro* culture attenuated *B. bovis* strain Att-S74-T3Bo, followed by superinfection with the homologous virulent strain Vir-S74-T3Bo, we identified novel correlates of protection against acute babesiosis and revealed critical aspects of the infection caused by attenuated and virulent parasites. After Att-S74-T3Bo inoculation, we observed signs of acute infection in all calves characterized by marginal clinical signs and a marked increase in monocytes with concomitant decrease in neutrophils and CD4^+^ T cells in peripheral blood. These alterations in the kinetics of monocytes, neutrophils, and CD4^+^ T cells coincided with a significant increase in serum concentration of TNFα, IFNγ, CXCL10, and IL-10 early after infection. Data indicate that infection with this attenuated *B. bovis* strain elicited both innate and acquired immune responses, which protected the animals against superinfection with a homologous virulent strain. In **Figure 7**, we provide a synopsis of the present data and propose a model for the immunological and clinical events following Att-S74-T3Bo infection in naïve cattle (**Figure 7A**), Vir-S74-T3Bo superinfection in Att-S74-T3Bo-protected cattle (**Figure 7B**), and Vir-S74-T3Bo infection in naïve cattle (**Figure 7C**). Collectively, the results demonstrate that Att-S74-T3Bo infection induces early activation of immune responses, corroborating and expanding results from previously published studies in *B. bovis* (19–22).

Cells of the monocyte/macrophage linage have been shown to play an important role in protection against apicomplexan parasites, such as *Babesia* spp. and *Plasmodium* spp., among other parasite species (46–49). The present data reinforce this concept, considering the pronounced monocytosis in Att-S74-T3Bo-infected calves that were protected against superinfection with a homologous virulent parasite strain. Even though the early monocytosis observed in peripheral blood of protected animals is novel, it is important to highlight that this is in agreement with previous results in the bovine spleen and underscores the importance of early activation of myeloid cells in the development of protective immune responses against *B. bovis* (19, 23). The functional relevance of the observed monocytosis in peripheral blood is currently unknown; however, we speculate that a host and/or parasite factor(s) may induce monocytopoiesis in the bone marrow early during infection. Subsequently, these monocytes may migrate from the bone marrow to the peripheral blood and then to the spleen, playing a role as phagocytic cells and a source of cytokines and oxidation reagents that control parasite growth. Noteworthy, the one control calf that did not succumb to Vir-S74-T3Bo infection also developed neutropenia and monocytosis starting at days 10 and 16 post-infection, respectively, indicating a delay compared to protected Att-S74-T3Bo-inoculated animals. Also, an interesting result of our study was the CD4^+^ T cell lymphopenia observed at day 7 after Att-S74-T3Bo infection. It is possible that after infection, CD4^+^ T cells migrate from peripheral blood to the spleen, a major organ during infection with blood pathogens, such as *Babesia* (50–52). This recruitment of CD4^+^ T cells to the spleen early after infection may be important, considering that this T cell population is associated with protection against *Babesia* infection (22, 53). We observed no alterations in CD8^+^, γδ, and B cells in peripheral blood after Att-S74-T3Bo and Vir-S74-T3Bo infections. Our results on the dynamics of CD4^+^ and CD8^+^ T cells after *B. bovis* infection differ from previous observations following infection with an *in vitro* culture attenuated *B. bigemina* strain (54). This discrepancy may be due to differences in the pathogenesis of babesiosis caused by *B. bovis* and *B. bigemina*, and/or distinct biological characteristics of the attenuated parasite strains used in the two studies. In addition, we found that increase in *B. bovis* parasite load did not affect the number of reticulocytes in peripheral blood, despite the reduction of RBC in infected animals. This contrasts with data showing that high parasitemia of *B. microti in vivo* in peripheral blood is prevented by an increase in the number of reticulocytes, underlining a significant biological difference between *B. bovis* and *B. microti* (53, 55).

Initial innate immune events elicited by encountering *Babesia* parasites may define the magnitude, quality, and outcome of the infection. It has been previously described that early activation of innate immune responses in the spleen is associated with protection against acute bovine babesiosis (19–22, 56, 57). Here, we demonstrated that Att-S74-T3Bo infection induces alterations in innate immune myeloid and lymphoid cells in peripheral blood, eliciting significant levels of the pro-inflammatory cytokines TNFα, CXCL10, and IFNγ in sera as early as 3 days post-infection. Interestingly, the regulatory cytokines IL-10 and IL-4 were also detected during acute infection with Att-S74-T3Bo, which implies that this attenuated strain induced protective responses characterized by a balanced profile of pro- and anti-inflammatory cytokines in peripheral blood. Considering the absence of significant expression of pro-inflammatory cytokines in sera of naïve animals infected with Vir-S74-T3Bo compared to calves inoculated with Att-S74-T3Bo, it is possible to speculate that virulent *B. bovis* parasites may sabotage the bovine immune system, as previously demonstrated in the mouse model during *B. microti* infection (58). This premise is also supported by the lack of alterations in the kinetics of immune cells and the absence of antibodies to *B. bovis* antigens after infection of naïve animals that either succumbed to acute infection or survived the acute phase of the disease. In that context, it is also possible to infer that there is currently no evidence that the cattle immune system is involved in the pathogenesis of acute bovine babesiosis caused by *B. bovis*. This potential escape from and/or downregulation of the immune system provoked by virulent *B. bovis* contrasts with findings in other apicomplexan parasites that induce overwhelming responses that are associated with immunopathogenesis (45, 59–61). Further studies are needed to understand the molecular components of virulent *B. bovis*, in comparison to attenuated strains, that may be responsible for subverting the immune system of cattle, leading to delayed immune responses and pathogenesis.

Attenuation of *Babesia* parasites by *in vitro* cultivation has been demonstrated before (54, 62–64), and we have previously reported that the *in vitro* culture attenuated *B. bovis* strain Att-S74-T3Bo is a non-clonal parasite line with lower genetic diversity compared to its homologous virulent strain (38). This genetic alteration in Att-S74-T3Bo has likely occurred during the long-term *in vitro* cultivation period of this strain and may be responsible for its attenuated phenotype; however, the actual mechanism of attenuation remains unknown. The present study demonstrates that animals inoculated with Att-S74-T3Bo developed mild signs of acute disease and a significantly lower parasitemia compared to naïve animals infected with Vir-S74-T3Bo. Yet, this observation cannot solely explain the attenuated phenotype of the strain, considering the alterations in immune blood cells and elicitation of immune responses following Att-S74-T3Bo infection. In previous studies, we showed that cattle infected with either 2x10^8^ or 10^7^ Att-S74-T3Bo parasites presented mild signs of acute disease and recovered without treatment (37, 38). Here in the present work, we demonstrated that calves infected with a lower parasite dose (10^6^ iRBC/calf) were not only infected but also developed protective immune responses against a homologous virulent *B. bovis* strain. Even though the attenuated phenotype of Att-S74-T3Bo has been confirmed by our work, additional studies are needed to investigate the molecular genetic mechanisms involved in attenuation of this strain compared to homologous and heterologous virulent parasites. Such mechanisms may involve the ability of virulent parasites to adhere to capillaries of vertebrates and/or express immune modulators. Also, it remains to be determined why naïve animals do not respond or present a delayed response to virulent *B. bovis* parasites. The relevance of this question is highlighted in our study where two naïve control animals that succumbed to acute disease on days 13 and 14 post-Vir-S74-T3Bo infection showed no alterations in the level of immune cells and cytokine profile compared to animals previously infected with attenuated parasites.

It has been demonstrated that antibodies against *B. bovis* antigens can neutralize the parasite growth *in vitro* and that passive transfer of sera from immune animals can protect naïve cattle from acute babesiosis (65–68). However, it is still unclear if the humoral immune response plays a role in protecting naïve animals from clinical signs of acute disease during primary infection. Here we show that all three naïve control calves developed severe acute disease, while two of them succumbed to acute infection, the third survived Vir-S74-T3Bo infection. Considering that the control calf that was able to control acute disease after Vir-S74-T3Bo inoculation without developing detectable antibodies, it is reasonable to speculate that humoral immune response is not essential for protection against a primary infection with a virulent parasite strain. Yet, it is possible that antibodies may play a critical role in protection against development of acute disease upon reinfection, as demonstrated in our study where Att-S74-T3Bo-infected calves, which developed significant levels of antibodies to parasite antigens, where fully protected against Vir-S74-T3Bo. The absence of significant alterations in the kinetics of immune cells and in the profile of cytokine expression in Att-S74-T3Bo-infected animals after Vir-S74-T3Bo superinfection also suggests that antibodies may play a role in protection upon reinfection. Additional studies are needed to evaluate the importance of the humoral immune response during primary and subsequent infections with *B. bovis*, and to identify novel antigens and/or epitopes that may be relevant for the elicitation of protective antibodies.

Understanding the immunological mechanisms induced by infection with attenuated *B. bovis* strains that protect cattle against acute disease upon primary infection and subsequent exposure to the parasite is critical to reveal immune correlates of protection and mechanisms of pathogenesis. This knowledge may lead to the rational design of vaccines to control bovine babesiosis. In that respect, based on the present data, we conclude that calves infected with Att-S74-T3Bo develop early activation of immune responses characterized by monocytosis, neutropenia, lymphopenia of CD4^+^ T cells, and a balanced expression profile of pro- and anti-inflammatory cytokines in peripheral blood (**Figure 7**). Strikingly, Att-S74-T3Bo-infected animals were protected against Vir-S74-T3Bo. Therefore, the immunological alterations in peripheral blood presented in this study can be considered correlates of protection against bovine babesiosis caused by *B. bovis*. The identification of novel immune markers of protection against *B. bovis* acute infection represents a step forward in our understanding of the protective mechanisms involved in bovine babesiosis. Based on the results, we propose that prospective vaccine trials should focus on live attenuated parasites, genetically modified live parasites, *Babesia* antigens, and novel adjuvants to induce early activation of myeloid cells, such as monocytes and neutrophils, and CD4^+^ T cells. In addition, future anti-*Babesia* vaccine evaluations should consider the development of a balanced profile of pro- and regulatory cytokines, especially TNFα, CXCL10, IFNγ, IL-10, and IL-4 in peripheral blood as a correlate of protection against acute disease. Considering the development of protective immunity in calves after infection with Att-S74-T3Bo, further investigations are needed to address the potential efficacy of this attenuated strain in preventing acute babesiosis in highly susceptible adult cattle and in eliciting protection against heterologous strains of *B. bovis*. Collectively, the findings of this study are relevant and represent a significant step toward the development effective vaccines against bovine babesiosis.

## Data availability statement

The original contributions presented in the study are included in the article/**Supplementary Material**. Further inquiries can be directed to the corresponding authors.

## Ethics statement

The animal study was reviewed and approved by University of Idaho Institutional Animal Care and Use Committee.

## Author contributions

Conception and design: RB, CS. Collection and assembly of data: RB, JL, SO, CS. Data analysis and interpretation: RB, JL, SO, HA, NT, MU, CS. Manuscript writing: RB, JL, SO, HA, NT, MU, CS. Final approval of manuscript: All authors.

## Funding

We acknowledge financial support from the USDA National Institute of Food and Agriculture (NIFA) (Award Number: 2020-67015-31809; Proposal Number: 2019-05375, Accession Number: 1022541), the International Development Research Center (IDRC) (Livestock Vaccine Innovation Fund (Grant 108525), funded by the Canadian Government and the Bill and Melinda Gates Foundation), and the United States Department of Agriculture (ARS-USDA CRIS 2090- 32000-040-00-D).

## Acknowledgments

We wish to express our gratitude to Paul Lacy, Jinna Navas, and Megan Jacks for their excellent technical assistance and animal care.

## Conflict of interest

The authors declare that the research was conducted in the absence of any commercial or financial relationships that could be construed as a potential conflict of interest.

## Publisher’s note

All claims expressed in this article are solely those of the authors and do not necessarily represent those of their affiliated organizations, or those of the publisher, the editors and the reviewers. Any product that may be evaluated in this article, or claim that may be made by its manufacturer, is not guaranteed or endorsed by the publisher.

## Supplementary material

Supplementary Figure 1 Immunoblots using sera from cattle infected with the *in vitro* culture attenuated *B. bovis* strain Att-S74-T3Bo (n=3) and the virulent *B. bovis* strain Vir-S74-T3Bo (n=3). DPI, days post-infection. DPSI, days post-superinfection. nRBC, normal red blood cells. M, molecular weight in kilodaltons (KDa).
