# Supplementary material for: Identification of novel immune correlates of protection against acute bovine babesiosis by superinfecting cattle with in vitro culture attenuated and virulent Babesia bovis strains
Source: Front Immunol. 2022 Nov 18;13:1045608. doi: 10.3389/fimmu.2022.1045608 (PMC9716085; doi:10.3389/fimmu.2022.1045608)
Supplement: Supplementary file 1 [file Table_1.docx]

Table S1. List of monoclonal antibodies used in this study to bovine CD14^+^ monocytes, CD335^+^ NK cells, CD4^+^ T cells, CD8^+^ T cells, γδ T cells, and B cells.

| Monoclonal Antibody | Isotype | Specificity | Source |
| --- | --- | --- | --- |
| CAM36A | IgG1 | CD14 monocytes | WSU Monoclonal Center |
| AKS1 | IgG1 | CD335 NK cells | Bio-Rad |
| ILA11A | Ig2a | CD4 T cells | WSU Monoclonal Center |
| 7C2B | Ig2a | CD8 T cells | WSU Monoclonal Center |
| GB21A | IgG2b | γδ T cells | WSU Monoclonal Center |
| BAQ155A | IgG1 | B cells | WSU Monoclonal Center |

Table S2. List of secondary reagents used in this study for flow cytometric analysis.

| Antibody Identification | Source |
| --- | --- |
| Alexa Flour™ 488 goat anti-mouse IgG1 | Thermo Fisher Scientific |
| Alexa Flour™ 647 goat anti-mouse IgG1 | Thermo Fisher Scientific |
| Alexa Flour™ 647 goat anti-mouse IgG2a | Thermo Fisher Scientific |
| Alexa Flour™ 647 goat anti-mouse IgG2b | Thermo Fisher Scientific |
| Alexa Flour™ 647 goat anti-mouse IgG | Thermo Fisher Scientific |
