# Supplementary figures and images for: Identification of novel immune correlates of protection against acute bovine babesiosis by superinfecting cattle with in vitro culture attenuated and virulent Babesia bovis strains
Source: Front Immunol. 2022 Nov 18;13:1045608. doi: 10.3389/fimmu.2022.1045608 (PMC9716085; doi:10.3389/fimmu.2022.1045608)

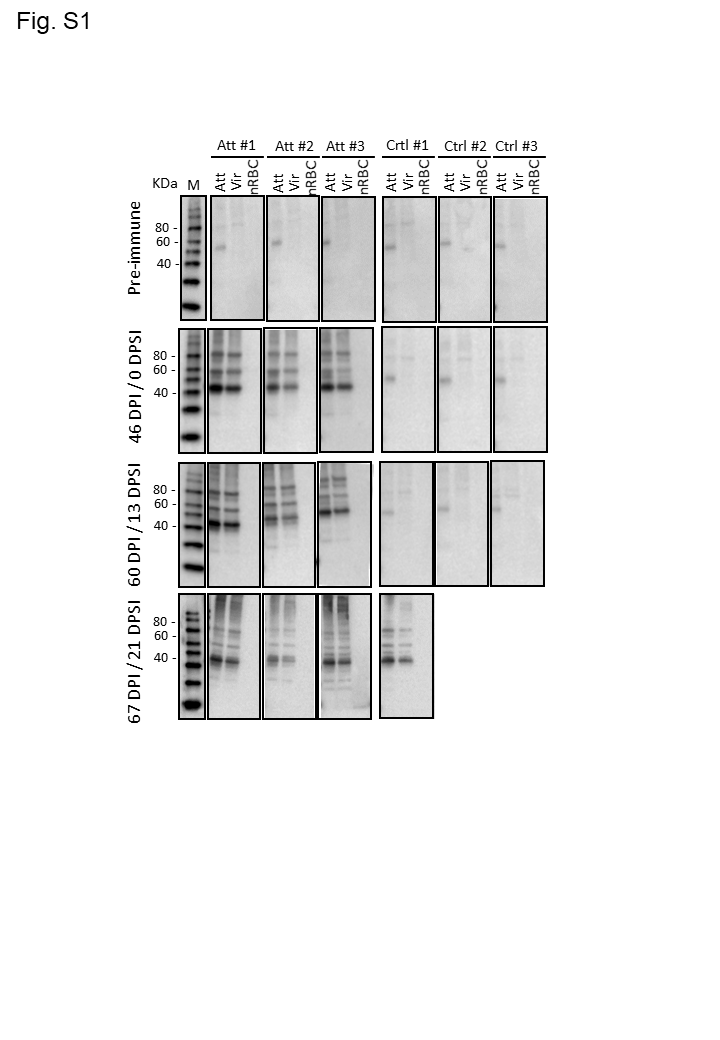

Supplement: Supplementary Figure 1 — Immunoblots using sera from cattle infected with the in vitro culture attenuated B. bovis strain Att-S74-T3Bo (n=3) and the virulent B. bovis strain Vir-S74-T3Bo (n=3). DPI, days post-infection. DPSI, days post-superinfection. nRBC, normal red blood cells. M, molecular weight in kilodaltons (KDa). [file Image_1.tif]
